# Repressing effect of transformed ginsenoside Rg3-mix against LPS-induced inflammation in RAW264.7 macrophage cells

**DOI:** 10.1186/s43141-023-00462-4

**Published:** 2023-01-19

**Authors:** Zuneera Marium, Muhammad Zubair Siddiqi, Ji-Hye Lee, Wan-Taek Im, Seong-Gu Hwang

**Affiliations:** 1 Department of Animal Life and Environmental Sciences, Hankyong National University, 327 Jungang-ro, Anseong-si, Gyeonggi-do 17579 Republic of Korea; 2 Department of Biotechnology, Hankyong National University, 327 Jungang-ro, Anseong-si, Gyeonggi-do 17579 Republic of Korea; 3 AceEMzyme Co., Ltd., Room 403, Academic-Industry Cooperation, 327 Jungang-ro, Anseong-si, Gyeonggi-do 17579 Republic of Korea; 4 HK Ginseng Research Centre, Hankyong National University, 327 Jungang-ro, Anseong-si, Gyeonggi-do 17579 Republic of Korea

**Keywords:** Ginsenosides, GRg3-mix, Macrophage RAW264.7, Anti-inflammation

## Abstract

**Background:**

Rg3-ginsenoside, a protopanaxadiol saponin, is a well-known adaptogen used for the prevention of cancer and inflammation. However, despite its distinct biological activity, the concentration of Rg3 in the total ginseng extract is insufficient for therapeutic applications. This study aims to convert PPD-class of major ginsenosides into a mixture of minor ginsenoside, to analyze its immune-regulatory role in macrophage cells.

**Results:**

Using heat and organic acid treatment, three major ginsenosides, Rc, Rd, and Rb1, were converted into a mixture of minor ginsenosides, GRg3-mix [Rg3(*S*), Rg3(*R*), Rg5, and Rk1]. Purity and content analysis of the transformed compound were performed using thin-layer chromatography (TLC) and high-performance liquid chromatography (HPLC), compared with their standards. Preceding with the anti-inflammatory activity of GRg3-mix, lipopolysaccharide (LPS)-stimulated murine RAW264.7 macrophage cells were treated with various concentrations of GRg3-mix (6.25, 12.5, 25, and 50 μg/mL). The cell viability assay revealed that the level of cell proliferation was increased, while the nitric oxide (NO) assay showed that NO production decreased dose-dependently in activated RAW264.7 cells. The obtained results were compared to those of pure Rg3(*S*) ≥ 98% (6.25, 12.5, and 25 μg/mL). Preliminary analysis of the CCK-8 and NO assay demonstrated that GRg3-mix can be used as an anti-inflammatory mediator, but mRNA and protein expression levels were evaluated for further confirmation. The doses of GRg3-mix significantly suppressed the initially upregulated mRNA and protein expression of inflammation-related enzymes and cytokines, namely inducible nitric oxide synthase (iNOS), cyclooxygenase-2 (COX-2), nuclear transcription factor kappa B (NF-κB), tumor necrosis factor (TNF-α), and interleukins (IL-6 and IL1B), as measured by reverse transcription-polymerase chain reaction and western blotting.

**Conclusions:**

Our pilot data confirmed that the mixture of minor ginsenosides, namely GRg3-mix, has high anti-inflammatory activity and has an easy production procedure.

## Background

**Fig. 1:**
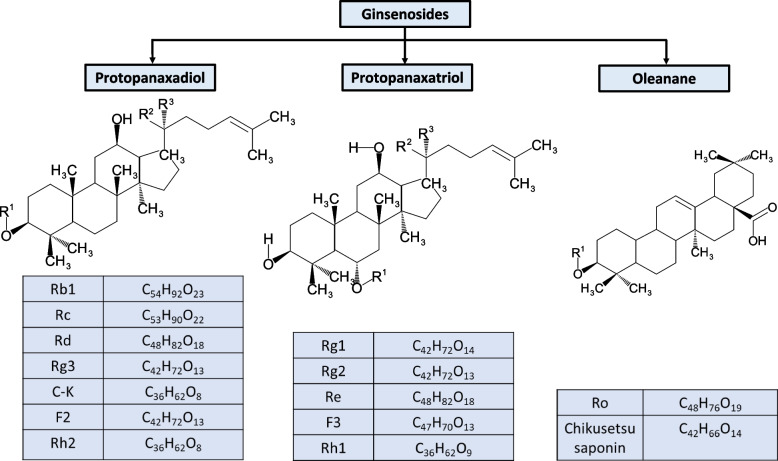
Structural classification of ginsenosides

*Panax ginseng* (family Araliaceae), also known as the “king of herbs,” is a well-known natural and traditional medicine in eastern Asia [1]. Its root consists of organic (80–90%) and inorganic (10%) substances, along with several active saponins, nitrogenous substances, carbohydrates, peptides, amino acids, vitamins, essential oils, and minerals [2]. Among them, ginsenosides are the principal bioactive components and are triterpene saponins that are used as marker compounds for ginseng species [3]. Ginsenosides are categorized into three broad classes based on their hydroxyl group position: protopanaxadiols (PPD), protopanaxatriols, and oleananes [4], as shown in Fig. 1. To date, nearly 200 major and minor ginsenosides have been reported, all of which have a steroidal structure [5]. Because of this structure, they can easily interact with cell membranes, intracellular and extracellular receptors, and membrane-bound ion channels, leading to alterations at the transcriptional level [6]. Based on the percentage of the total ginseng extract, ginsenosides can be divided into two categories: major ginsenosides and minor ginsenosides. Major ginsenosides account for 80% of the total ginseng extract, including Rc, Rd, Rb2, Rb1, Rg1, and Re, whereas minor ginsenosides are present in the trace amounts ≤ 0.2%, including 20(*S*) and 20(*R*)- Rg3, Rg5, Rh2, Rk1, Rh1, and Rg2 [7–9].

The interest of researchers in the medicinal use of ginseng is increasing owing to its pharmacological effects, which has led to the publication of various studies on its principal active components and their efficacy [10]. The amount of rare minor ginsenosides in the total ginseng extract is insufficient to meet clinical and industrial demands [11]. Therefore, minor ginsenosides (such as F2, Rg3(*S*), Rg3(*R*), Rg5, Rk1, and CK) are derived from the major ginsenosides using conversion processes such as biotransformation, chemical or physical conversion, and heat acid treatment [12, 13]. Moreover, studies have demonstrated that absorption of major ginsenosides by the gastrointestinal tract is difficult and the gut microbiome deglycosylases major ginsenosides and ultimately converts them into minor ginsenosides [14, 15].

Prior studies have demonstrated the regulatory role of minor ginsenosides in inflammation [16], cancer [17], obesity [18], diabetes [19], immune stimulation [20], vasodilation [21], and neuroprotection [22], indicating their therapeutic potential. Among the known minor ginsenosides, Rg3 is particularly effective in blocking inflammatory responses [23, 24]. In one study, Rg3 inhibited Th2 cytokine and eotaxin production to repress inflammation and oxidative stress by lowering AHR, eosinophil infiltration, and mucus hypersecretion [25]. The protective and therapeutic potential of Rg3 was demonstrated by the inhibition of oxidants in APAP-induced hepatic damage by alleviating apoptosis and necrosis [26]. The anti-scarring property of Rg3 was demonstrated by the modulation of NF-ĸB/I-ĸB signaling to reduce hypertonic scar formation in rabbit ears [27]. The immune-protective effects of Rg3 and its molecular mechanism in the PI3K/AKT/mTOR pathway have also been reported [28]. Additionally, Rg3 has been shown to protect in vitro expression of arginase-1 in intoxicated peritoneal macrophages to resolve inflammation [29]. Nonetheless, ginsenoside Rk1 has been reported to inhibit LPS-stimulated NF-κB and Jak2/Stat3 pathways in macrophages [30]. In another study, Rk1 inhibited NF-κB and IκB activation, thereby suppressing inflammation [31]. Regarding the anti-inflammatory effects of ginsenoside Rg5, a study proved that Rg5 treatment suppressed NF-κB, iNOS, and COX-2 in HepG-2 cells against hepatitis [32]. Moreover, the protective effects of Rg5 ginsenoside against kidney injury by inhibition of NLRP3 inflammasome activation in a diabetic mouse confirmed its potency to suppress inflammation [33].

To the best of our knowledge, the influence and efficacy of a mixture of transformed minor ginsenosides processed by heat and organic acid treatment of major ginsenosides have rarely been reported. The present study aimed to provide heat and organic acid treatment to the PPD-class of major ginsenosides (Rc, Rd, and Rb1) and transform them into a mixture of minor ginsenosides, that is, the GRg3-mix (Rg3(*S*), Rg3(*R*), Rk1, and Rg5), to evaluate its anti-inflammatory role in LPS-activated RAW 264.7 murine macrophages. This study compares the anti-inflammatory effects of the transformed GRg3-mix with those of the extracted Rg3(*S*) (≥ 98% pure) through a series of cellular reactions, including cell cytotoxicity assay, nitric oxide assay, and mRNA and protein expression levels. Our results will help to investigate the dose-dependent effects of GRg3-mix on LPS-stimulated inflammatory cytokines in RAW264.7 macrophages and the underlying molecular mechanism of these effects. The findings of this study will augment the application of transformed ginsenosides in the pharmaceutical and functional food industries, thereby replacing the conventional procedures of extracting minor ginsenosides, which are expensive and time-consuming.

## Methods

### Composition of transformed GRg3-mix

The saponins of Korean ginseng, that is, the PPD-class of major ginsenosides, including Rc, Rd, and Rb1, were purchased from AceEMzyme Co., Ltd., Anseong, South Korea. In the preparation of the GRg3-mix, organic acid and heat treatment were applied to the PPD-class of major ginsenosides to transform it into GRg3-mix, as described previously [34]. A 50-g/L PPD-class of major ginsenosides (Rc, Rd, and Rb1) were dissolved in 2% w/v citric acid with distilled water, followed by heating for 15 min at 121°C. Ginsenoside conversion was confirmed using thin-layer chromatography (TLC) and high-performance liquid chromatography (HPLC) analyses. In preparation for a stock solution of GRg3-mix, 0.1% dimethyl sulfoxide (DMSO) was used, and Dulbecco’s modified Eagle’s medium (DMEM) was used for further experimental dilutions.

### Identification and purity analysis of transformed GRg3-mix

Silica gel plates 60F_254_ (Merck, Germany) were used for thin-layer chromatography (TLC) analysis with a solvent chloroform-methanol-water (CHC_l3_-CH_3_OH-H_2_O) in the ratio of 65:35:10. Ginsenoside standards were used as a marker to identify GRg3-mix spots (Rg3(*S*), Rg3(*R*), Rk1, and Rg5). For the visualization of spots, 10% (v/v) H_2_SO_4_ was sprayed on the TLC plates, after which they were heated for 5 min at 110°C. For high-performance liquid chromatography (HPLC) of GRg3-mix, an HPLC system (Younglin Co. Ltd, Korea) with a quaternary pump, automatic injector, and single wavelength UV detector (model 730D) was used. Younglin AutoChro 3000 software was used for peak detection and integration. Prodigy ODS (2) C18 column (5 μm, 150 × 4.6 mm i.d.; Phenomenex, USA) with a guard column (Eclipse XDB C18, 5 μm, 12.5 × 4.6 mm i.d.) was used to perform the separation. The mobile phases used were B (water) and C (acetonitrile). The gradient elution started with 68% solvent B and 32% solvent C; the flow rate was kept at 1.0 mL/min. The absorbance was measured at 203 nm with an injection volume of 25μl for 28 min [35].

### Preculturing of RAW264.7 macrophages

Murine macrophage cells (RAW264.7) from the Korean Cell Line Bank, Seoul, South Korea, were precultured in DMEM (Gibco^TM^, South Korea) augmented with 10% heat-incapacitated fetal bovine serum (FBS, Gibco^TM^, South Korea), 1% Pen/Strep (Gibco^TM^, South Korea), and 3.7 mg/mL of NaHCO_3_ in an incubator at 37 °C with 5% CO_2_ until the cells reached 70% confluency.

### Cytotoxicity assessment of GRg3-mix

The cells were seeded in a microtiter plate (1×10^5^ cells/mL) for overnight surface attachment. The following day, they were treated with different doses of GRg3-mix (0, 6.25, 12.5, 25, 50, and 100 μg/mL) and Rg3(*S*) (0, 6.25, 12.5, and 25 μg/mL), respectively, and incubated for an hour at 37 °C with 5% CO_2_. The cells were then induced with 10 μg/mL lipopolysaccharide (LPS, L3129 Sigma-Aldrich) and incubated again for 48 h at 37 °C with 5% CO_2_. For a stock solution of 1 mg/mL LPS, 10 mg lyophilized LPS was dissolved in 10 mL sterile PBS and further aliquoted into the desired working concentration of 10 μg/mL. After 48 h, a CCK-8 reagent (0.001 g/L, Dojindo, Japan) was added to the treated cells. Optical density was measured at 450nm using an ELISA plate reader (Tecan, Switzerland). The proliferation of the treated cells was calculated as the percentage of control cells.

### Nitric oxide determination

To determine the NO produced by the cells, a clean bench with 70% confluent cells was seeded at a concentration of 1×10^5^ cells/mL in a microtiter plate for overnight attachment. The next day, GRg3-mix (0, 6.25, 12.5, 25, and 50 μg/mL) or Rg3(*S*) (0, 6.25, 12.5, and 25 μg/mL) treatment was administered to the attached cells, along with LPS (10 μg/mL) induction for 48 h. After which, 50 μL supernatant was collected from the treated cells and used for NO determination along with 50μL Griess reagent (Sigma-Aldrich, South Korea). An ELISA microplate reader (Tecan, Switzerland) was used to measure the absorbance at 540 nm. NO produced by the treated cells was expressed as a percentage of control cells.

### RNA extraction

A density of 1×10^5^ cells/mL was seeded in a microplate with 6 sample wells and incubated overnight at 37°C with 5% CO_2_. The attached cells were treated with the desired concentrations of GRg3-mix (0, 6.25, 12.5, 25, and 50 μg/mL). After an hour, LPS (10 μg/mL) activation of macrophages was performed, and the cells were incubated for a further 48 h. Total RNA was isolated from the treated cells using the RNAiso Plus kit (Takara Shuzo Co., Japan) according to the manufacturer’s instructions.

### Reverse transcription-polymerase chain reaction (RT-PCR)

cDNA was synthesized using a Revert Aid first-strand cDNA synthesis kit (Thermo Scientific, USA) with a final concentration of 250 ng/μL of the extracted RNA. The final volume of the resultant mixture was incubated at 65 °C for 5 min, followed by an ice shock for 1 min. The reaction mixture (Thermo Fisher Scientific, South Korea) containing 40 U/μL Riboblock-RNAse-inhibitor, 10 mM dNTPs-mix, 200 U/L transcriptional yeast-RevertAid, 5× reaction buffer with MgCl_2_ for DNase-I (10×), and 0.2 μg/mL random Hexa-primer was added and incubated for 5 min at 25 °C followed by incubation at 42 °C for 60 min. The termination reaction was performed at 95°C for 5 min in a thermal circulator (Bio-Rad C1000^TM^ Thermal Cycler), and the synthesized cDNA was immediately stored at −20°C.

RT-PCR was performed using a premix (Maxime^TM^ RT-PCR kit, South Korea) according to the manufacturer’s instructions. The oligonucleotide sequences of the primers (Cell Signaling Technology, USA) are shown in Table 1 and were used to quantify the following genes: NF-κB, cyclooxygenase-2, inducible nitric oxide synthase (iNOS), tumor necrosis factor-alpha (TNF-α), interleukin-1 beta (IL1 β), interleukin 6 (IL-6), and beta-actin (β-actin) [36]. An initial denaturation of 2 min at 95°C was followed by the following cycle parameters: 1-min annealing of NF-κB (57°C), COX-2 (54°C), iNOS (65°C), TNF-α (57°C), IL-1β (60°C), IL-6 (55°C), and β-actin (56°C) was tailed by 1 min of elongation at 72°C and 1 min of denaturation at 94°C. A 2% agarose gel was used for the separation of the resulting PCR products, while ethidium bromide stained the bands for visualization. All reactions were performed in triplicate. The results were analyzed by comparing the quantification cycle (Cq) value of the genes to that of β-actin.

**Table 1:** Oligonucleotide sequence of primers

Name	Primer sequences		bp
β-actin	Sense	5′-CACCCCAGCCATGTACGT-3′	201
	Antisense	5′-GTCCAGACGCAGGATGGC-3′	
iNOS	Sense	5′-AATGGCAACATCAGGTCGGCCATCACT-3′	807
	Antisense	5′-GCTGTGTGTCACAGAAGTCTCGAACTC-3′	
COX-2	Sense	5′-GGAGAGACTATCAAGATAGT-3′	721
	Antisense	5′-ATGGTCAGTAGACTTTTACA-3′	
NF-κB	Sense	5′-GGCCTGCAAAGGTTATCGTT-3′	300
	Antisense	5′-TGTCTGTGAGTTGCCGGTCTT-3′	
IL-6	Sense	5′ CATGTTCTCTGGGAAATCGTG-3′	340
	Antisense	5′-AACGCACTAGGTTTGCCGAGTA-3′	
TNF-α	Sense	5′-ATGAGCACAGAAAGCATGATC-3′	351
	Antisense	5′-TACAGGCTTGTCACTCGAATT-3′	
IL1-β	Sense	5′-TGCAGAGTTCCCCAACTGGTACATC-3′	387
	Antisense	5′-GTGCTGCCTAATGTCCCCTTGAATC-3′	

### Western blotting

The cells were seeded at a concentration of 1×10^5^ cells/mL in a six-well microplate and incubated overnight with 5% CO_2_ at 37°C. The next day, the attached cells were treated with different concentrations of GRg3-mix (0, 6.25, 12.5, 25, and 50 μg/mL) and induced with LPS (10 μg/mL). After 48 h, 200 μL of protein extraction solution (PRO-PREP^TM^ 17081, intron-biotechnology, South Korea) was used to extract protein from the cells, followed by a modified Bradford assay to determine the protein concentration. Sodium dodecyl sulfate-polyacrylamide gel electrophoresis (SDS-PAGE) was used to separate the protein (30 μg each), after which samples were transferred to a nitrocellulose membrane (Tech & Innovation). The nitrocellulose membranes were blocked with 5% skim milk, followed by hybridization with the following primary antibodies procured from Abcam^TM^, South Korea: rabbit-polyclonal anti-NF-κB (ab16502), mouse-monoclonal anti-β actin (ab8226), rabbit-polyclonal anti-COX2 (ab15191), rabbit-polyclonal anti-iNOS (ab3523), mouse-monoclonal anti-IL-6 (ab9324), and mouse-monoclonal anti-TNFα (ab1793). After incubation with horseradish-peroxidase-conjugated secondary antibodies (AB-Frontier-LFQC0103, South Korea), an enhanced chemiluminescence reagent (Westsave Gold, AbFrontier, South Korea) was used to soak the membranes for 5 min, followed by exposure to the radiographic film (Agfa-HealthCare). Protein expression levels were normalized to those of a β-actin control.

### Statistical analysis

All experiments were conducted in triplicates, and the results were expressed as the mean ± standard deviation. Duncan’s multiple range test and one-way analysis of variance were performed to evaluate the differences between the control group and GRg3-mix- treated cells. Statistical software (SPSS 19.0, SPSS Inc., Chicago, IllinoisL, USA) pointed out statistically significant values, with *p* < 0.05.

## Results

### Composition and identification of GRg3-mix

**Fig. 2:**
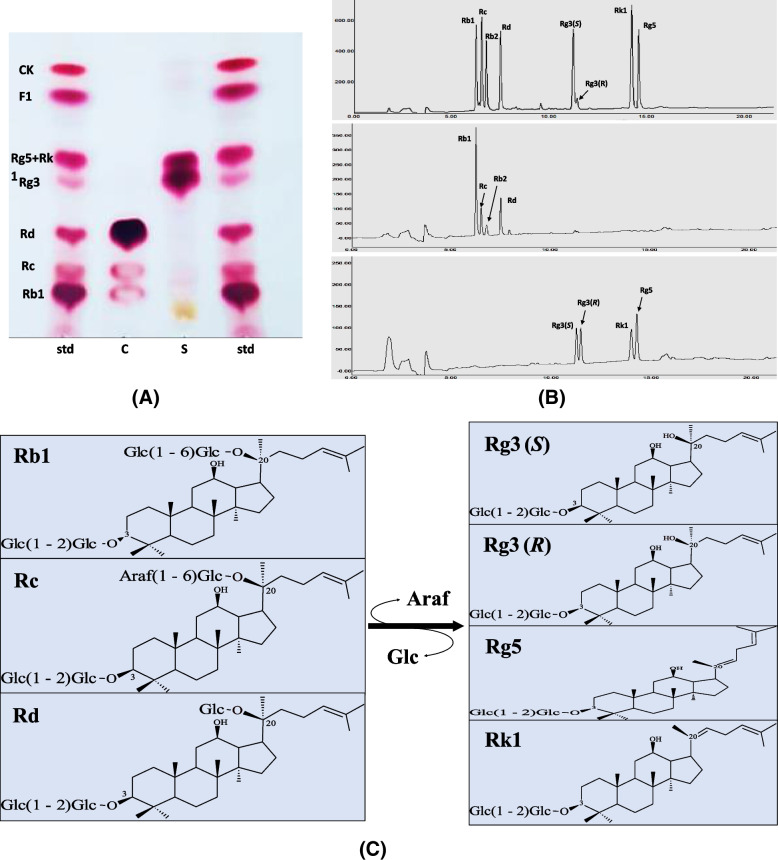
Conversion of PPD-mix ginsenosides into Rg3-mix ginsenosides. **A** TLC and **B** HPLC analysis of heat acid-transformed GRg3-mix, for purity and content check by using ginsenoside standards to compare spots and peaks obtained. **C** Transformation pathway of major PPD-class of major ginsenosides into minor ginsenoside Rg3-mix [Rg3(*S*), Rg3(*R*), Rg5 and Rk1]. std, ginsenoside standard; C, control; S, sample

The PPD-class of major ginsenosides (Rc, Rd, and Rb1) was efficiently transformed into GRg3-mix (Rg3(*S*), Rg3(*R*), Rg5, and Rk1) by heat and organic acid treatment, as shown in Fig. 2. TLC analysis indicated two spots of GRg3-mix when the results were compared with the ginsenoside standard. The upper spot contained Rg5+Rk1, while the lower spot contained Rg3(*S*) and Rg3(*R*), as shown in Fig. 2A. TLC results were further confirmed by HPLC peaks marked in comparison with the HPLC standard, as shown in Fig. 2B. The transformation pathway of the major PPD-class of major ginsenosides into the minor ginsenoside Rg3-mix after the heat and organic acid treatment is depicted in Fig. 2C.

### Effect of GRg3-mix and Rg3(S) on RAW 264.7 cell proliferation

**Fig. 3:**
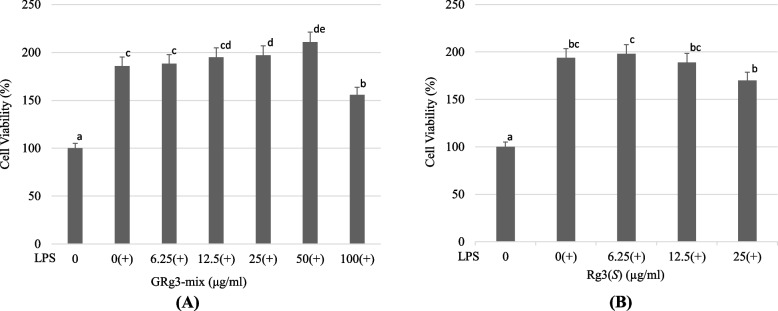
Effect of GRg3-mix and Rg3(*S*) on cell viability of LPS-activated RAW264.7 macrophages. LPS-induced RAW264.7 macrophages were incubated with different concentrations of GRg3-mix (**A**) and Rg3(*S*) (**B**), to determine cell viability using CCK-8 assay. All values are presented as mean ± SD (*n*=3). Different superscripts on the bars point out significant differences (*p* < 0.05). LPS, lipopolysaccharide; SD, standard deviation

To determine the cytotoxicity of GRg3-mix and the proliferation of RAW 264.7, macrophages were assessed using a CCK-8 reagent, and the results were compared with those of Rg3(*S*). The results showed that cell viability increased gradually with an increase in the concentration of GRg3-mix (Fig. 3A). At concentrations ranging from 6.25 to 50 μg/mL, cell viability increased by approximately 30–50%, after which a decline in cell proliferation was observed. These results suggest that GRg3-mix has a dose-dependent proliferative effect on murine RAW 264.7 macrophage cells when compared to the control group (naïve) (*p* < 0.05). On the other hand, cell viability increased in Rg3(*S*)-treated cells up to 6.25 μg/mL; however, higher concentrations of Rg3(*S*), i.e., 12.5–25 μg/mL showed decreased cell proliferation (Fig. 3B), indicating that pure Rg3(*S*) had the highest activity at lower concentrations. However, the cell viability assay results showed that the GRg3-mix has promising anti-inflammatory activity up to 50 μg/mL.

### Effect of GRg3-mix and Rg3(S) on NO production by RAW 264.7 cells

**Fig. 4:**
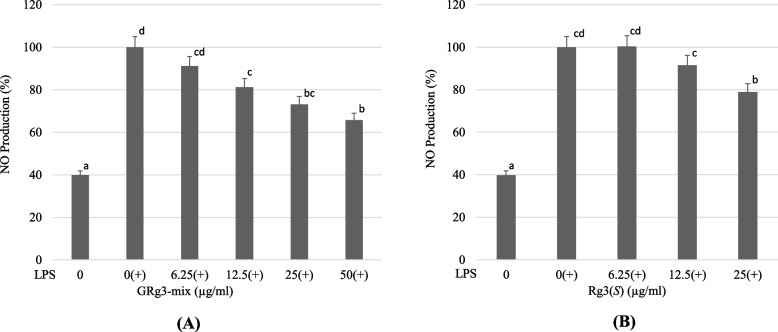
Effect of GRg3-mix and Rg3(*S*) on NO production in LPS-stimulated RAW264.7 macrophages. The LPS-induced RAW264.7 macrophages were incubated with different concentrations of GRg3-mix (**A**) and Rg3(*S*) (**B**) to determine the level of NO production using the Griess reagent. All values are presented as mean ±SD (*n*=3). Different superscripts on the bars point out significant differences (*p* < 0.05). LPS, lipopolysaccharide; NO, nitric oxide; SD, standard deviation

A Griess reagent kit was used to measure NO production in LPS-induced RAW264.7 cells. A significant increase in NO production was observed upon stimulation with LPS, but GRg3-mix treatment (6.25–50 μg/mL) decreased the production of NO dose-dependently (Fig. 4A). Decreased levels of nitric oxide in LPS-induced RAW 264.7 macrophages to support the fact that GRg3-mix plays a role in suppressing nitric oxide-induced inflammation. Similarly, Rg3(*S*) treatment (6.25–25 μg/mL) decreased NO production dose-dependently (Fig. 4B), proving its anti-inflammatory role. However, the effect of Rg3(*S*) on NO production was steady and less significant than that of the GRg3-mix. These results further support our hypothesis that GRg3-mix is an invasive compound to regulate inflammation.

### Effect of GRg3-mix on mRNA expression of inflammatory cytokines

**Fig. 5:**
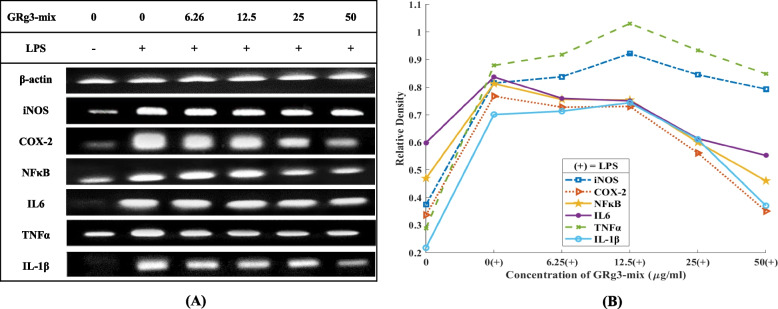
Effects of GRg3-mix on mRNA expression of LPS-induced RAW 264.7 cells. RAW264.7 macrophages were treated with various concentrations of GRg3-mix (6.25, 12.5, 25, and 50μg/mL) followed by LPS induction. Total RNA was extracted, using an RNAiso Plus kit, after 48 h. RT-PCR was performed to visualize the mRNA expression of inflammatory cytokines (**A**). Bands were visualized using ImageJ software (**B**). All values are presented as mean ± SD (*n*=3). RT-PCR, real-time polymerase chain reaction; LPS, lipopolysaccharide; SD, standard deviation

The results from the preliminary analysis of CCK-8 and NO production assays were further confirmed by evaluating the mRNA expression of the marker inflammatory cytokines. As LPS induced the mRNA expression of iNOS, NF-κB, IL-6, TNF-α, COX-2, and IL-1β, RT-PCR was performed to visualize their expression. LPS stimulation substantially upregulated gene expression, which was dose-dependently downregulated by treatment with GRg3-mix (6.25–50 μg/mL) (Fig. 5A, B). The results proved that GRg3-mix suppressed LPS-induced inflammatory cytokines by transcriptionally inhibiting their expression.

### Effect of GRg3-mix on protein expression of inflammatory cytokines

**Fig. 6:**
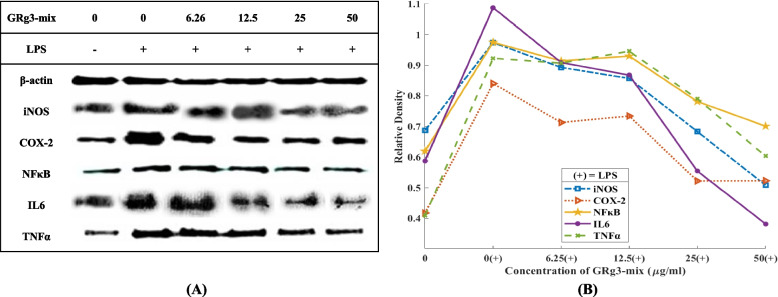
Effects of GRg3-mix on protein expression of LPS-induced RAW 264.7 macrophages. RAW264.7 cells were treated with GRg3-mix (6.25, 12.5, 25, and 50μg/mL) followed by LPS activation. After 48 h of incubation, the protein was extracted and quantified using the Bradford assay. SDS-PAGE was performed to visualize the protein expression of inflammatory cytokines (**A**). Bands were visualized using ImageJ software (**B**). All values are presented as mean ± SD (*n*=3). LPS, lipopolysaccharide; PES, protein extraction solution; SD, standard deviation

Protein expression in LPS-induced RAW264.7, treated with GRg3-mix, was found to correlate with mRNA expression. All protein-expressing inflammatory cytokines (iNOS, NF-κB, IL-6, TNF-α, and COX-2) were downregulated upon GRg3-mix (6.25–50 μg/mL) treatment for 48 h. Initially, LPS stimulation upregulated the expression of inflammatory mediators. However, it was gradually downregulated with different concentrations of GRg3-mix (Fig. 6A, B).

## Discussion

Ginseng is considered a panacea on the Korean Peninsula and consists of various advantageous compounds [37]. Ginsenosides are used to cure all bodily ailments. Scientific advancements have enabled us to explore the biological effects and molecular principles of major or derived minor ginsenosides [38]. Many studies have summarized the efficacy and usefulness of ginseng extract as a whole or as a single extracted ginsenoside [39, 40]. However, there is no study available to prove the in vitro anti-inflammatory effect of ginsenoside mixture (transformed by heat and organic acid treatment); therefore, we performed this study to understand the effect of GRg3-mix in murine RAW 264.7 macrophage cells.

Inflammation is a non-specific immune reaction in response to bodily grievance [41]. LPS can cause inflammation and activate macrophages via pathogen-associated molecular patterns (PAMPs). Hence, macrophages recognize PAMPs by Toll-like receptors, stop proliferation, and activate the biosynthesis of inflammatory mediators, such as interleukins (ILs), TNF-α, and NF-κB pathway, which generates co-stimulatory molecules required for the adaptive immune response against foreign invasion [42]. Owing to their importance in immune scrutiny, macrophages are of great importance for the study of the NF-κB pathway [43].

NO is a gaseous moiety released by macrophages, monocytes, and neutrophils as a defense mechanism against foreign invasion [44]. Prolonged NO emission can stop the normal functioning of the tissue, leading to its degradation [45]. iNOS is an inducible isoform of NOS that catalyzes NO production and is activated in a calcium-independent manner. Excessive NO production is positively correlated with upregulated iNOS expression and vice versa [46]. In this study, we showed that GRg3-mix treatment (6.25–50 μg/mL) significantly downregulated iNOS expression in LPS-induced murine macrophages. In addition, preliminary analysis of the CCK-8 and NO assays also showed that the GRg3-mix has a notable role in mediating the anti-inflammatory response. Hence, this study demonstrated the anti-inflammatory role of GRg3-mix with its key components [Rg3(*S*), Rg3(*R*), Rg5, and Rk1].

Prostaglandin E2 (PGE2) is a primary mediator of inflammation, produced by COX-2 [47]. The inducible enzyme “COX-2” is considered the most popular drug target for studying the “commencement and resolution of inflammation,” due to its upregulation in prolonged inflammation [48]. In the current study, the downregulation of mRNA and protein expression of COX-2 suggests that GRg3-mix is a potent compound for the commencement and resolution of inflammation.

**Fig. 7:**
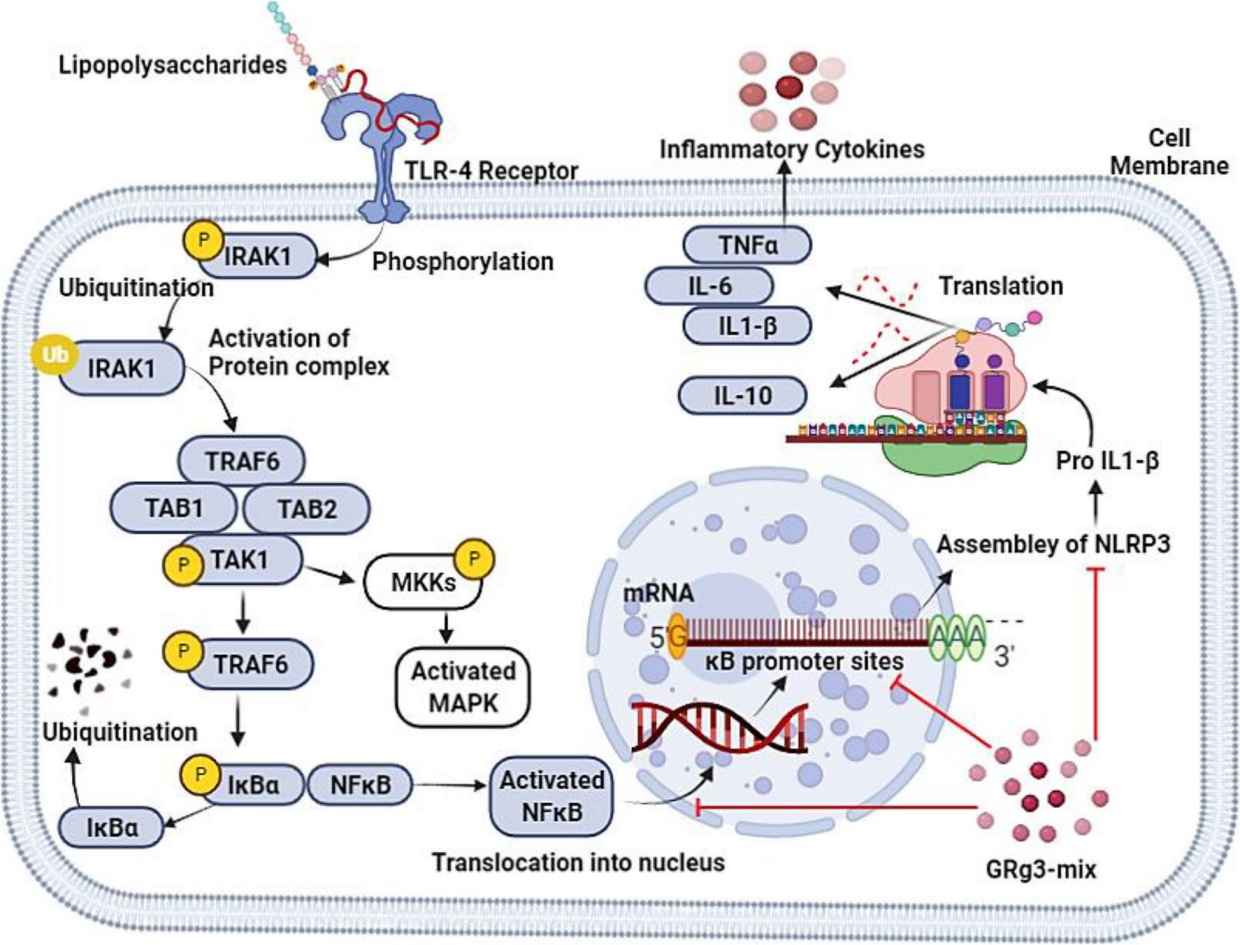
Induction and stimulation of NF-κB signaling pathway in response to LPS and a possible mechanism of action of GRg3-mix against LPS activity

NF-κB and its family of transcription factors play a key role in innate immunity and inflammation studies. NF-κB has five proteins in its family, namely, NFκB1 (p50), NFκB2 (p52), Rel-A (p65), Rel-C, and Rel-B. These proteins form homodimer and heterodimer complexes and their activity is controlled by two major pathways: (1) classical/canonical activation and (2) alternative/noncanonical pathways [49]. Macrophages initiate an innate immune response against bacterial pathogens by phagocytosis, thereby eradicating them with the help of reactive oxygen species [50, 51]. TLR-4 is the main mediator of LPS signaling, as it recognizes pathogen-associated molecular patterns, such as bacterial strains, and initiates phosphorylation of interleukin-1 receptor-associated kinases (IRAK-1) [52]. Phosphorylated IRAK-1 is degraded by ubiquitination to activate multimers of the protein complex (TAB1, TAB2, TRAF6, and TAK1). Activated TAK-1 initiates the phosphorylation of IKKs and MKKs [53]. These IKKs play a role in the phosphorylation of IκB-α, which is an inhibitor of NF-κB. In the cytoplasm, the NF-κB p50/p65 complex exists in an inactivated form bound to IκB. Phosphorylation of IκBα leads to ubiquitination and consequent degradation of IκB-α by proteasomes. This degradation sets NF-κB free and translocates it to the nucleus, where it attaches to specific promoter sequences. Activated NF-κB initiates transcription of inflammatory mediators, cytokines, and chemokines (Fig. 7). Hence, increased secretion of ILs and TNF occurs, which provokes an innate immune response [54]. In contrast, activated MKKs phosphorylate and stimulate members of the JNK/p38 MAP kinase (MAPK) family [55].

In the alternate/noncanonical pathway, IKKα is phosphorylated by NF-κB inducing kinase (NIK), which results in the advancement of P100 into mature-P52. Subsequently, NF-κB2 (P52) translocates into the nucleus in the form of a dimer along with RelB, where RelB stimulates promoter activity and P52 pairs up with the specific κB sites of the promoter sequences of the inflammatory cytokines iNOS, COX-2, ILs, and TNFα by stimulating the survival pathway [56]. In this study, the increase in mRNA and protein expression levels of NF-κB in response to LPS stimulation in RAW264.7 macrophage cells was dose-dependently downregulated upon pre-treatment with different concentrations of GRg3-mix. From these findings, we assume that GRg3-mix suppresses inflammatory cytokine expression via the NF-κB-mediated pathway in LPS-activated macrophage cells. In accordance with previous findings [57] and the results of the current study, we deduce that GRg3-mix has a regulatory role on the inflammatory markers and MAPK/NF-κB pathways, either by inhibiting LPS-induced IKK-β phosphorylation or by IRAK activation. However, more research is required to completely understand the mechanism of action of the GRg3-mix and to identify the exact target site of this transformed steroidal compound.

## Conclusions

Consequently, research has defined the biological activities of Rg3 ginsenosides, including anti-inflammation, but most studies have reported the efficacy of a single and purified Rg3 compound. The extraction and purification of minor ginsenosides (Rg3) from ginseng is a multistep, time-consuming, and money-consuming process. Using a mixture of major ginsenosides to produce minor ginsenosides with strong anti-inflammatory activity is a promising way to reduce the time, cost, and work procedure. In the present study, the PPD-class of major ginsenosides (Rc, Rd, and Rb1) was converted into a mixture of minor ginsenosides (Rg3(*S*), Rg3(*R*), Rg5, and Rk1) by heat and organic acid treatment, which showed a promising ability to combat inflammation. Compared to Rg3(*S*), GRg3-mix treatment efficiently increased cell viability and decreased NO production in LPS-induced murine RAW264.7 macrophages. In addition, it efficiently downregulated the mRNA and protein expression of the inflammatory cytokines TNF-α, iNOS, NF-κB, IL-6, COX-2, and IL-1β. In conclusion, the economically produced GRg3-mix has promising anti-inflammatory activity with a simple production procedure as compared to pure and extracted Rg3(*S*) compounds.

## Data Availability

The datasets generated during and/or analyzed during the current study are available from the corresponding author on reasonable request.
